# Characterization of gut microbiota in Vietnamese children under 5 years of age with acute and persistent diarrhea: A cross-sectional study

**DOI:** 10.1097/MD.0000000000050433

**Published:** 2026-08-28

**Authors:** Nghi Huu Chung, Tuan Anh Nguyen, Van Hung Pham, Luan Thanh Nguyen, Nhan Trong Phan, Chuyen Quoc Vo, Nguyen Thanh Nhu

**Affiliations:** a Faculty of Medicine, Can Tho University of Medicine and Pharmacy, Can Tho, Vietnam; b Department of Pediatrics, School of Medicine, University of Medicine and Pharmacy, Ho Chi Minh, Vietnam; c Department of Internal Medicine and Gastro-Hepato Integrated Research Team (GHIRT-002.TCM2025), School of Medicine, University of Medicine and Pharmacy at Ho Chi Minh City, Ho Chi Minh, Vietnam; d Nam Khoa Biotek Company Limited, Ho Chi Minh, Vietnam; e Department of Laboratory, Gene Solutions, Ho Chi Minh, Vietnam; f Children’s Hospital 1, Ho Chi Minh, Vietnam.

**Keywords:** children, diarrhea, gut microbiota, microbiota dysbiosis, Vietnam

## Abstract

Gut microbiota alterations have been increasingly associated with diarrheal diseases, a leading cause of morbidity and mortality in children under 5 years of age. However, explorations examining microbiota profiles across different diarrhea durations remain limited. Our study investigated microbiota alterations in Vietnamese children with diarrhea. We recruited 97 children under 5 years of age and divided them into 3 groups: healthy children (HC; n = 48), children with acute diarrhea (AD; n = 32), and children with persistent diarrhea (PD; n = 17). We collected and analyzed stool samples using 16S rRNA gene sequencing targeting the V3 to V4 region. Taxonomic classification was performed. Alpha diversity and beta diversity metrics were estimated. LEfSe analysis identified differentially abundant bacterial taxa across groups. Gut microbiota composition and diversity differed significantly among HC, AD, and PD. Alpha diversity tended to decline from HC to AD and was lowest in PD, with significant reductions observed for the Shannon index and Faith PD. Compared with HC, AD showed enrichment of Actinobacteriota and potentially pathogenic genera, including *Streptococcus* and *Escherichia–Shigella*, together with depletion of beneficial commensal taxa. PD exhibited more pronounced dysbiosis, including expansion of Proteobacteria, depletion of Bacteroidota-associated commensals, and a significantly reduced Bacteroidota-to-Firmicutes ratio. Children with AD and PD exhibited distinct gut microbiota alterations, with more pronounced microbial dysbiosis observed in PD. These findings improve our understanding of gut microbiota alterations associated with pediatric diarrhea and support future longitudinal studies incorporating comprehensive pathogen identification to clarify temporal relationships, evaluate microbiome-based biomarkers, and explore microbiota-targeted interventions.

## 1. Introduction

Diarrheal diseases remain a significant cause of morbidity and mortality among children under 5 years of age, particularly in developing countries.^[1]^ Despite significant advancements in reducing mortality related to diarrhea, this condition continues to account for approximately 446,000 diarrhea-related deaths among children younger than 5 years in 2016.^[2]^ Diarrhea is a prevalent gastrointestinal condition marked by the passage of loose or watery stools 3 or more times daily, particularly common among children.^[3]^ Acute diarrhea (AD) typically resolves within 2 weeks, whereas persistent diarrhea (PD), which lasts between 2 and 4 weeks,^[3]^ produces more severe health risks.^[1,4]^

Diarrheal episodes frequently disrupt the well-balanced gut microbiota, which is essential for maintaining intestinal homeostasis and optimal health.^[5]^ This disruption results in microbiota dysbiosis, characterized by reduced microbial diversity and an imbalance in bacterial communities, favoring pathogenic over commensal microorganisms. Microbiota dysbiosis compromises gut barrier function, impairs nutrient absorption, alters immune responses, induces systemic inflammation, and disrupts gut–organ communication.^[6–8]^ Emerging evidence suggests that microbiota dysbiosis may accompany diarrheal episodes and may also have long-term consequences, including persistent microbiota immaturity and impaired nutritional status.^[9,10]^

Some recent studies have explored the relationship between diarrhea and gut microbiota. The alteration of gut microbiota during diarrhea can be categorized into 3 stages: the early stage (days 1–5), the middle stage (days 7–10), and the late stage (after day 14).^[11,12]^ In the early phase of diarrhea, gut microbial richness and diversity are often reduced, whereas Proteobacteria and *Streptococcus* may become enriched.^[12,13]^ In contrast, there is a notable depletion of obligatory anaerobes such as *Blautia*, *Prevotella*, *Faecalibacterium,* Lachnospiraceae, and Ruminococcaceae.^[13]^ Although these overall dysbiotic patterns overlap across diarrheal etiologies, pathogen-associated differences are observed. Bacterial diarrhea is often linked to an increase in *Streptococcus* and oral-associated bacteria, whereas viral diarrhea tends to exhibit higher levels of *Bifidobacterium*.^[13]^ The middle stage is characterized by an increase in *Bacteroides* abundance, while the late stage shows a broader recovery with increased diversity and enrichment of Firmicutes and *Prevotella*.^[5,12]^ PD may be associated with various external and internal factors, but it is often considered to be multifactorial in individual patients.^[14]^ One hypothesis regarding gut microbiota alteration is that a subset of patients with AD fail to reestablish a normal microbial composition, and this prolonged dysbiosis may contribute to the development of PD.^[15]^ For instance, research conducted in Ethiopia identified significant differences in the gut microbiota of children with AD and PD compared to healthy controls (HCs), showing reduced bacterial diversity and an overrepresentation of pathogenic genera such as *Escherichia*, *Campylobacter*, and *Streptococcus.*^[16]^ Moreover, several studies have reported reduced gut microbiota diversity in children with diarrhea, while persistent diarrhea may be associated with more pronounced alterations in microbial composition and depletion of gut commensals.^[16,17]^ These findings highlight the role of gut microbiota in influencing the progression and outcomes of diarrheal diseases. However, data on gut microbiota alterations in children with PD remain very limited.

Despite the growing body of evidence on the gut microbiota in children with diarrhea,^[13]^ there is still limited information on Southeast Asian populations, particularly in Vietnam, where diarrhea remains a prevalent cause of pediatric hospitalization. Understanding the alterations in the gut microbiota associated with different diarrheal durations in Vietnamese children could provide valuable insights into the microbial mechanisms underlying diarrhea and identify potential targets for microbiota-targeted therapeutic strategies aimed at restoring microbial balance. Therefore, this study aims to characterize the gut microbiota diversity and composition in Vietnamese children under 5 years of age suffering from AD and PD in comparison with HCs. By comparing the microbial profiles across these groups, we seek to identify specific microbial signatures associated with diarrhea duration and explore the potential for microbiota-directed interventions to improve clinical outcomes in affected children.

## 2. Materials and methods

### 2.1. Study design and study population

This cross-sectional study used samples obtained from a larger pediatric research project conducted at Children’s Hospital 1 in Ho Chi Minh City. The parent project included several pediatric populations, comprising healthy children (HC) and children with AD, PD, community-acquired pneumonia, short bowel syndrome, or cholestatic jaundice. Eligible participants were children aged 2 months to under 5 years who either attended the outpatient clinic (HCs) or were hospitalized with diarrhea and met the eligibility requirements for enrollment. Children with diarrhea were defined according to the World Health Organization (WHO) guidelines as the passage of 3 or more loose or liquid stools per day.^[3]^ In this study, AD was characterized by diarrhea lasting <2 weeks, and PD as diarrhea lasting between 2 and 4 weeks. HC were children who attended routine health checkups and had no gastrointestinal symptoms or other illnesses within 14 days prior to enrollment. From April 2022 to September 2024, a total of 97 children participated in the study, including 32 children with AD, 17 children with PD, and 48 HCs. It should be noted that the 48 HC participants included in this analysis were the same control cohort used in a previous study of gut microbiota alterations in Vietnamese children with community-acquired pneumonia conducted within the same parent project.^[18]^ However, the present study addressed a distinct research question and involved different disease cohorts, comparisons, and analyses. None of the children with AD or PD were included in the community-acquired pneumonia cohort.^[18]^

Children were excluded from the study if their guardians did not provide informed consent. Furthermore, children with diarrhea were excluded if they had 1 or more concurrent acute or underlying diseases that could influence gut microbiota composition. Other exclusion criteria included antibiotic (Abx) or probiotic use within the preceding 3 months,^[19]^ with the exception of children in the PD group, because these children had frequently received such treatments before hospital admission. Exclusion was also applied to children with a history of allergies, congenital or hereditary disorders, or diarrhea associated with confirmed lactose intolerance or celiac disease. Finally, children who followed special diets (restrictive or vegetarian diets) or had incomplete essential data were excluded.

### 2.2. Clinical data collection

For each participant, clinical data were collected in a systematic and standardized manner using a unified case report form completed by study physicians. Demographic information, feeding practices, birth history, medication use (including Abx and probiotics), and illness duration were obtained through direct interviews with caregivers. Anthropometric measurements, including body weight, height (for children ≥24 months), or length (for children <24 months), were assessed and recorded. Nutritional status was assessed using WHO growth standards based on weight-for-height/length *Z*-scores (WHZ).^[20]^ Overweight was defined as a WHZ > + 2 standard deviations (SD) but < + 3 SD, while obesity was classified as a WHZ > + 3 SD. Acute malnutrition (wasting) was identified when the WHZ was less than −2 SD. Chronic malnutrition (stunting) was defined as a height/length-for-age *Z*-score of <−2 SD. Dehydration was classified according to WHO criteria into 3 categories: no dehydration, some dehydration, and severe dehydration, based on 4 main clinical signs: general condition, sunken eyes, thirst, and skin turgor.^[3]^ Stool consistency was assessed using the Lewis–Heaton stool scale,^[21]^ which classifies stools into 7 types: type 1 (separate hard lumps, like nuts), type 2 (sausage-shaped but lumpy), type 3 (like a sausage but with cracks on the surface), type 4 (like a sausage or snake; smooth and soft), type 5 (soft blobs with clear-cut edges), type 6 (fluffy pieces with ragged edges; mushy), and type 7 (watery, no solid pieces).

### 2.3. Sample collection

Each child provided 1 stool sample. For children with diarrhea, samples were collected at hospital admission (for AD, between days 1 and 5 of illness; and for PD, between days 14 and 28). For HC, stool samples were collected from the 1st bowel movement of the day. Approximately 1 g of stool was collected from each participant using the DNA/RNA Shield Stool Collection Kit (Zymo Research) to stabilize microbial DNA and RNA. Stool samples were transported to the genomic sequencing laboratory at Gene Solutions in Ho Chi Minh City within 48 hours of collection. DNA was subsequently extracted from the samples in DNA/RNA Shield tubes and stored at −80°C until sequencing.^[19,22]^

### 2.4. Microbiota analysis

#### 2.4.1. DNA extraction and preparation of 16S ribosomal RNA gene amplicon libraries

DNA was extracted from stool samples using the QIAamp Fast DNA Stool Mini Kit (QIAGEN), following the manufacturer’s protocol. The quality and concentration of DNA were determined using the Nanodrop 1000 spectrophotometer (Thermo Fisher Scientific). A DNA concentration of ≥25 ng/μL and purity (A260/A280) between 1.8 and 2.0 were required for downstream analysis. Samples not meeting these criteria were re-extracted until adequate quality was achieved.^[22]^

#### 2.4.2. Polymerase chain reaction

The V3 to V4 hypervariable region of the bacterial 16S rRNA gene was amplified using the following primers:^[23]^ Forward primer: 5′ TCGTCGGCAGCGTCAGATGTGTATA AGGACAGCCTACGGGNGGCWGCAG-3′ and reverse primer: 5′ GTCTCGTGGGCTCG GAGATGTGTATAAGAGACAGGACTACHVGGGTATCTAATCC-3′. The polymerase chain reaction (PCR) conditions were as follows: initial denaturation at 98°C for 30 seconds, followed by 25 cycles of 98°C for 10 seconds, 55°C for 20 seconds, and 72°C for 20 seconds, with a final extension at 72°C for 5 minutes.^[22]^ PCR products were visualized using electrophoresis. Indices were added during a 2nd PCR using the Nextera Index Kit V2 (Illumina). The PCR products were pooled and purified using magnetic beads.

#### 2.4.3. Sequencing and data analysis

Sequencing was performed on an Illumina MiSeq platform using the MiSeq Reagent Kit V3 (MS-102-3003; Illumina Inc.) for 2 × 300 bp paired-end sequencing.^[24,25]^ Raw FASTQ files generated from the Illumina MiSeq platform were quality-checked and analyzed using the Quantitative Insights Into Microbial Ecology 2 (QIIME 2; Northern Arizona University, Flagstaff) pipeline. All sequences were trimmed to achieve an average Phred quality score >30. The DADA2 plugin (Callahan Lab, North Carolina State University, Raleigh) was used to generate amplicon sequence variants (ASVs) and assign taxonomy to each representative ASV sequence using the SILVA database^[22,26]^ (version 138.1; Leibniz Institute DSMZ – German Collection of Microorganisms and Cell Cultures GmbH, Braunschweig, Germany).

Alpha diversity was assessed using the Shannon index, Pielou evenness, Chao1 index, and Faith phylogenetic diversity. The beta diversity was measured using Bray–Curtis distance, Jaccard distance, UniFrac distances and visualized with principal coordinates analysis and nonmetric multidimensional scaling plots.^[26,27]^ Linear discriminant analysis effect size (LEfSe) was used to identify the bacterial taxa differentially represented between groups at different taxonomic levels.^[28]^ A linear discriminant analysis (LDA) was used to estimate the effect size of each differentially abundant feature (LDA score ≥2). Other images were generated using the R (version 4.4.2; R Foundation for Statistical Computing, Vienna, Austria).

### 2.5. Statistical analysis

Categorical variables were summarized as frequencies and percentages and compared using the chi-square test or Fisher exact test, as appropriate. Continuous variables were presented as mean ± SD or median and interquartile range and compared using the independent-samples *t* test, Mann–Whitney *U* test, 1-way analysis of variance, or Kruskal–Wallis test, as appropriate. Beta diversity was assessed using distance matrices and permutational multivariate analysis of variance. Differences in relative abundance were estimated with 95% bootstrap confidence intervals. Multiple comparisons were adjusted using the Benjamini–Hochberg method. A 2-sided *P* value <.05 or adjusted *q* value <0.05 was considered statistically significant.

### 2.6. Ethics approval

This study was approved by the Institutional Review Board of Children’s Hospital 1, Ho Chi Minh City, Vietnam, on March 31, 2022 (approval number: 88/GCN-BVNĐ1). Written informed consent was obtained from the parents or legal guardians of all participants prior to enrollment. All procedures involving human subjects were conducted in accordance with the ethical standards of the institutional and national research committees and with the 1964 Helsinki Declaration and its later amendments or comparable ethical standards.

## 3. Results

### 3.1. Baseline characteristics

Table 1 presents the baseline characteristics of the 3 groups. There were no significant differences in age, sex, WHZ, or height-for-age *Z*-score among the groups (*P* > .05). In addition, birth mode and type of feeding were comparable across groups. Dehydration was observed in 18.8% of children with AD, and no child with diarrhea experienced severe dehydration requiring intensive care. Among the 17 children with PD, 9 (52.9%) had received Abx before hospital admission, including 6 who received cefixime and 3 who received azithromycin.

**Table 1:** Demographic and clinical characteristics of the 3 study groups.

Variable	HC (n = 48)	AD (n = 32)	PD (n = 17)	*P* value
Age (mo)				
Median (min, max)	7.6 (1.1, 47)	16.3 (4.0, 40.4)	6.9 (3.0, 59.9)	.120
Gender				
Female, n (%)	23 (47.9)	9 (28.1)	5 (29.4)	.146
WHZ, n (%)				
WHZ < −2 SD	5 (10.4)	4 (12.5)	1 (5.9)	.350
−2 SD < WHZ < +2 SD	35 (72.9)	25 (78.1)	16 (94.1)	
WHZ > +2 SD	8 (16.7)	3 (9.4)	0	
HAZ, n (%)				
HAZ < −2 SD	5 (10.4)	4 (12.5)	1 (5.9)	.770
HAZ > −2 SD	43 (89.6)	28 (87.5)	16 (94.1)	
Antibiotics*, n (%)	0	0	9 (52, 9)	–
Dehydration, n (%)	0	6 (18, 8)	0	–
Dysentery, n (%)	0	0	0	–
Probiotics†, n (%)	0	0	17 (100)	–
Birth mode, n (%)				
Vaginal delivery	31 (64.6)	19 (59.4)	7 (41.2)	.240
Cesarean section	17 (35.4)	13 (40.6)	10 (58.8)	
Type of feeding, n (%)				
Breast-feeding	20 (41.7)	15 (46.9)	9 (52.9)	
Formula	5 (10.4)	3 (9.4)	4 (23.5)	.370
Mix	23 (47.9)	14 (43.8)	4 (23.5)	
Lewis–Heaton scale, n (%)				
Type 3–4	48 (100)	0	0	
Type 6	0	15 (46.9)	10 (58.9)	–
Type 7	0	17 (53.1)	7 (41.1)	

AD = acute diarrhea, HAZ = height-for-age *Z*-score, HC = healthy control, PD = persistent diarrhea, SD = standard deviation, WHZ = weight-for-height *Z*-score.

* Antibiotic use before hospital admission.

† Probiotic use before hospital admission. Statistical comparisons among groups were performed using analysis of variance (continuous variables) and the chi-square test (categorical variables).

### 3.2. Diversity of gut microbiota among the 3 groups

Forward and reverse reads were merged, denoised, and checked for chimeras using DADA2. Subsequently, rare sequences (<0.1%) were filtered out, and sequences classified as mitochondria, chloroplast, archaea, or eukaryota were removed. This resulted in 11,111,470 high-quality sequences (median 113,557; minimum 1449; and maximum 186,131). A total of 10,670 features (ASVs) remained for downstream analyses of diversity and community composition.

**Figure 1.**
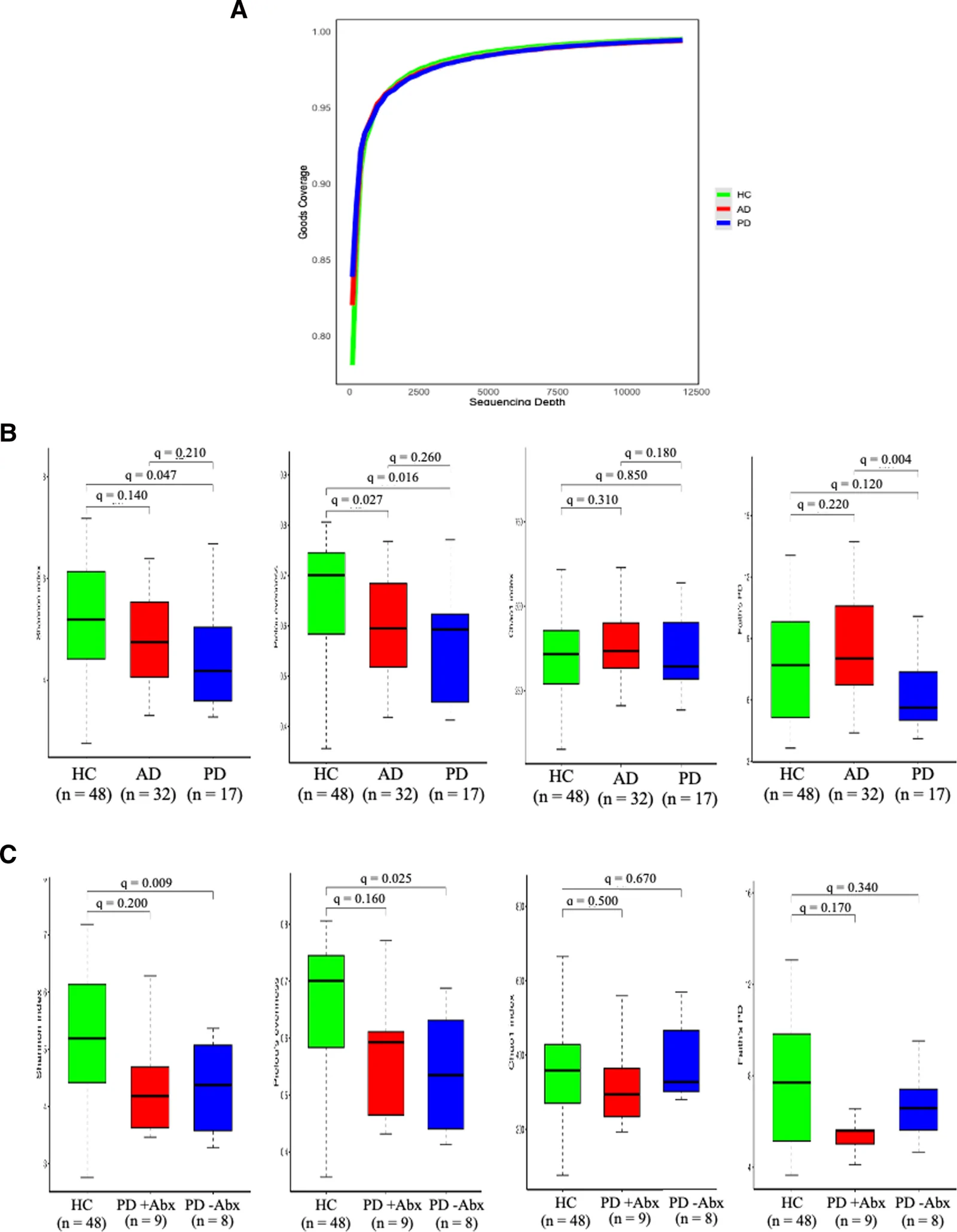
Alpha diversity of the gut microbiota among healthy children (HC), children with acute diarrhea (AD), and children with persistent diarrhea (PD). (A) Goods coverage rarefaction curves showing sequencing coverage across sequencing depths for the 3 groups, with coverage values approaching 1.0, indicating sufficient sequencing depth. (B) Comparison of alpha diversity among HC, AD, and PD using 4 complementary indices: Shannon index (community diversity), Pielou evenness (community evenness), Chao1 index (estimated richness), and Faith phylogenetic diversity (Faith PD). (C) Subgroup analysis stratified by antibiotic exposure, comparing HC with PD children who received antibiotics before stool collection (PD + Abx) and those who had not received antibiotics (PD − Abx). Pairwise comparisons were performed using the Kruskal–Wallis test with Benjamini–Hochberg correction; *q* values are shown above the boxplots. Abx = antibiotics.

Analysis of alpha diversity in 48 HC, 32 children with AD, and 17 children with PD showed that the Good coverage rarefaction curves approached 1.0 in all 3 groups, indicating sufficient sequencing depth and coverage for downstream analyses (Fig. 1A). Among the 4 alpha diversity indices, the Shannon index showed a progressive decrease from HC to AD and reached the lowest value in PD, with significantly lower diversity in PD than in HC (*q* = 0.047, Fig. 1B). Similarly, Pielou evenness decreased from HC to PD, with a significant difference between HC and PD (*q* = 0.016, Fig. 1B). In contrast, the Chao1 index showed no significant differences among the 3 groups (all *q* > 0.05, Fig. 1B). Faith phylogenetic diversity (Faith PD) was significantly lower in PD than in AD (*q* = 0.004), whereas no significant differences were observed between HC and AD or between HC and PD (Fig. 1B).

To evaluate the potential influence of Abx exposure, a subgroup analysis was performed by stratifying the PD group into children who had received Abx before stool collection (PD + Abx, n = 9) and those who had not (PD − Abx, n = 8) (Fig. 1C). The Shannon index was significantly lower in PD − Abx than in HC (*q* = 0.009), whereas no significant difference was observed between HC and PD + Abx (*q* = 0.200). Similarly, Pielou evenness was significantly lower in PD − Abx than in HC (*q* = 0.025), but did not differ significantly between HC and PD + Abx (*q* = 0.160). Chao1 and Faith PD showed no significant differences between HC and either PD subgroup (all *q* > 0.05). These findings indicate that the reductions in Shannon diversity and evenness observed in PD remained significant only in the subgroup without prior Abx exposure.

**Figure 2.**
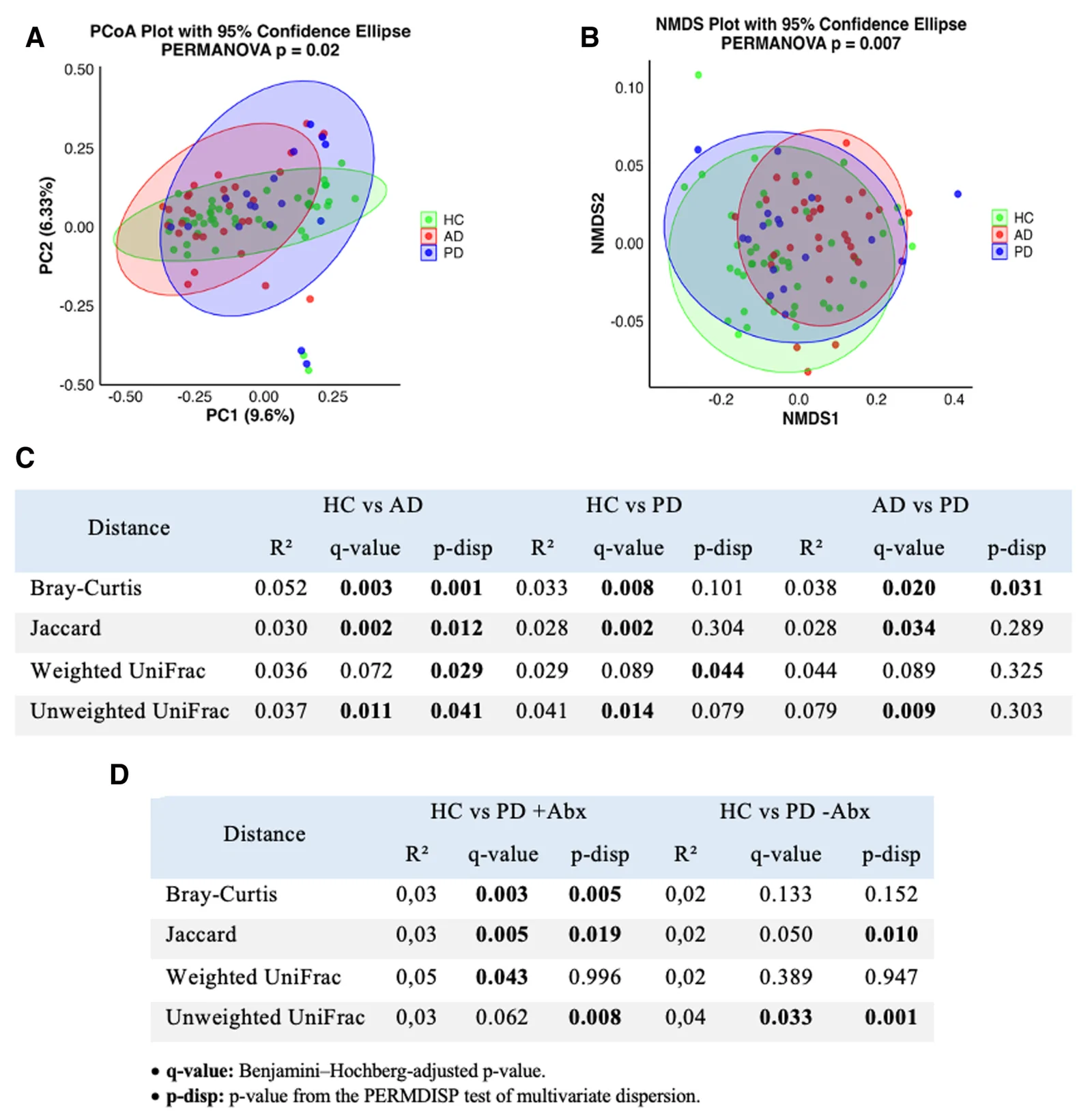
Beta diversity of the gut microbiota among healthy children (HC), children with acute diarrhea (AD), and children with persistent diarrhea (PD). (A) Principal coordinates analysis (PCoA) based on Bray–Curtis distance with 95% confidence ellipses, showing overall differences in microbial community structure among the 3 groups. (B) Nonmetric multidimensional scaling (NMDS) based on Jaccard distance with 95% confidence ellipses, demonstrating significant differences in microbial community composition among groups. (C) Pairwise comparisons of beta diversity among HC, AD, and PD using Bray–Curtis, Jaccard, weighted UniFrac, and unweighted UniFrac distance metrics. For each comparison, the coefficient of determination (*R*^2^), Benjamini–Hochberg-adjusted *P* value (*q* value), and PERMDISP *P* value (*P* disp) are presented. (D) Subgroup analysis stratified by antibiotic exposure, comparing HC with children with persistent diarrhea who received antibiotics before stool collection (PD + Abx) and those who had not received antibiotics (PD − Abx). Beta diversity was evaluated using the same 4 distance metrics, and *R*^2^, *q* values, and PERMDISP *P* values are reported. Abx = antibiotics, PERMDISP = permutational multivariate analysis of dispersion.

Beta diversity analysis revealed significant differences in gut microbiota composition among the 3 study groups. Principal coordinates analysis based on Bray–Curtis distance (Fig. 2A) and nonmetric multidimensional scaling (Fig. 2B) showed partially distinct but overlapping microbial community structures among the 3 groups, with significant overall differences detected by permutational multivariate analysis of variance (*P* = .020 and *P* = .007, respectively).

Pairwise comparisons using multiple beta diversity metrics further supported these findings (Fig. 2C). Bray–Curtis and Jaccard distances revealed significant differences between HC and both AD and PD (all *q* ≤ 0.008), as well as between AD and PD (*q* = 0.020 and q = 0.034, respectively). Unweighted UniFrac analysis also demonstrated significant differences between HC and AD (*q* = 0.011), HC and PD (*q* = 0.014), and AD and PD (*q* = 0.009). In contrast, weighted UniFrac distances did not differ significantly between any pair of groups after Benjamini–Hochberg correction (all *q* > 0.05). Permutational multivariate analysis of dispersion analysis indicated that the differences in beta diversity distance metrics between HC and AD could be explained, at least in part, by differences in within-group dispersion (*P* disp < 0.05). In contrast, the differences in beta diversity between HC and PD, as well as between AD and PD, were primarily attributable to differences in microbiota composition rather than within-group dispersion, except for the Bray–Curtis distance in the comparison between AD and PD (*P* disp = 0.031) (Fig. 2C).

To evaluate the potential influence of Abx exposure, a subgroup analysis was performed by stratifying the PD group into children who had received Abx before stool collection (PD + Abx) and those who had not (PD − Abx) (Fig. 2D). Compared with HC, the PD + Abx subgroup remained significantly different based on Bray–Curtis (*q* = 0.003), Jaccard (*q* = 0.005), and weighted UniFrac (*q* = 0.043) distances, whereas no significant difference was observed for unweighted UniFrac (*q* = 0.062). For the PD − Abx subgroup, a significant difference from HC was observed for unweighted UniFrac distance (*q* = 0.033), while Jaccard distance was of borderline statistical significance (*q* = 0.050). Bray–Curtis and weighted UniFrac distances were not significantly different (*q* = 0.133 and q = 0.389, respectively). However, the significant permutational multivariate analysis of dispersion results for Bray–Curtis and Jaccard in the HC versus PD + Abx comparison and for Jaccard and Unweighted UniFrac in the HC versus PD − Abx comparison indicate that some observed subgroup differences may be partly attributable to unequal within-group dispersion. Overall, stratification by prior Abx exposure resulted in distinct beta diversity patterns in children with PD; however, these subgroup findings should be interpreted cautiously because unequal within-group dispersion was observed for some distance metrics.

### 3.3. Gut microbiota composition among the 3 groups

**Figure 3.**
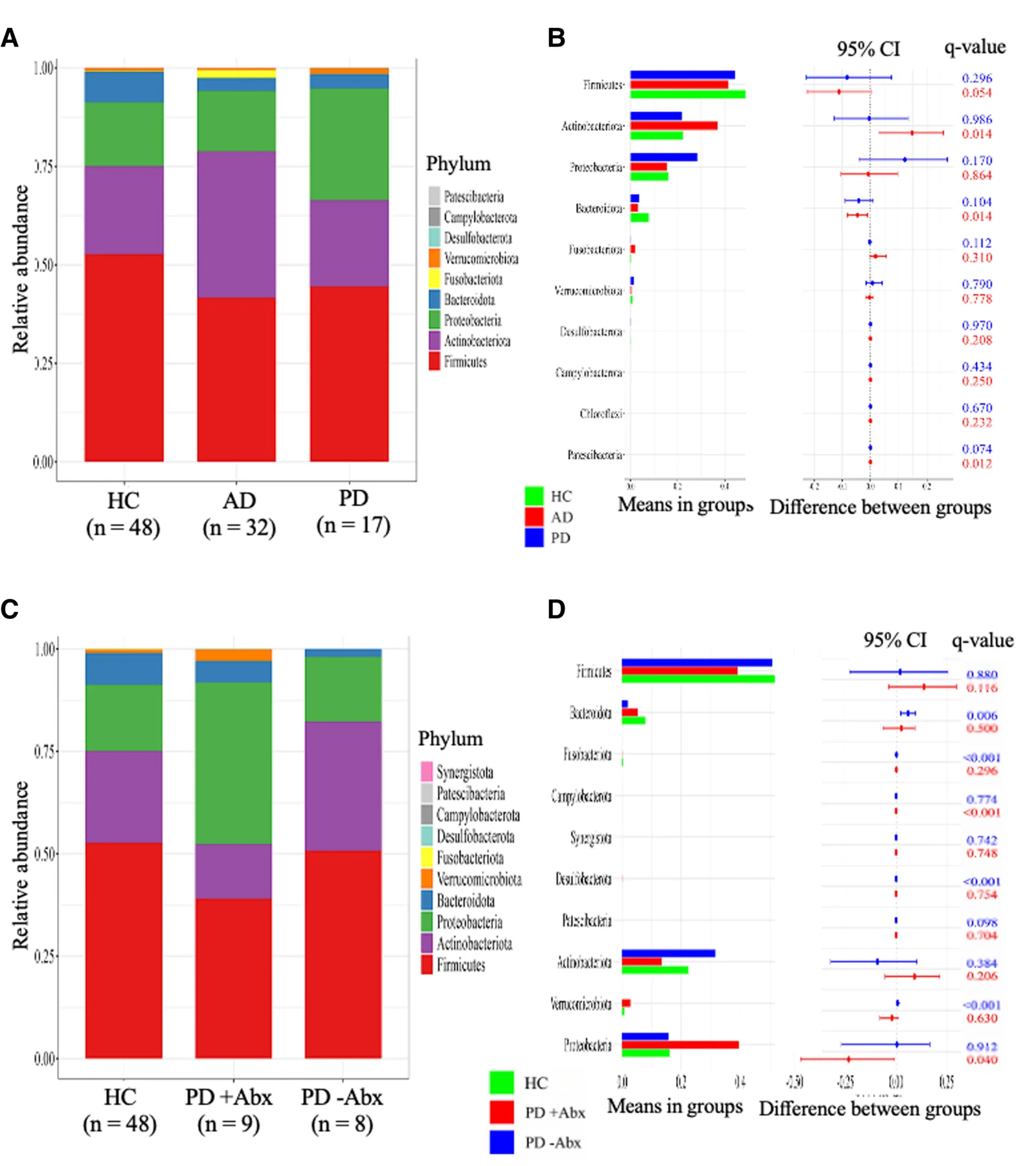
Phylum-level composition of the gut microbiota and subgroup analysis according to antibiotic exposure. (A) Relative abundance of the predominant bacterial phyla in healthy children (HC), children with acute diarrhea (AD), and children with persistent diarrhea (PD). (B) Mean relative abundance of the predominant bacterial phyla in each group and forest plots showing the differences in relative abundance between AD and HC (red) and between PD and HC (blue), with 95% confidence intervals (95% CI) and Benjamini–Hochberg-adjusted *P* values (*q* values). (C) Relative abundance of the predominant bacterial phyla in HC, children with persistent diarrhea who received antibiotics before stool collection (PD + Abx), and those who had not received antibiotics (PD − Abx). (D) Mean relative abundance of the predominant bacterial phyla and forest plots comparing PD + Abx and PD − Abx with HC, showing the differences in relative abundance with 95% confidence intervals (95% CI) and *q* values. Abx = antibiotics.

After excluding ASVs unclassified at the phylum level, 12 phyla, 54 classes, 83 orders, 148 families, 375 genera (30 unclassified), and 830 species (238 unclassified) were identified. Gut microbiota composition varied significantly among the 3 groups at both the phylum and genus levels. At the phylum level, 4 phyla made up the majority in 3 groups. These included Firmicutes (overall: 46.41%; HC: 52.53%; AD: 42.62%; and PD: 45.04%), followed by Actinobacteriota (overall: 27.13%; HC: 23.73%; AD: 35.98%; and PD: 21.83%), Proteobacteria (overall: 19.94%; HC: 14.18%; AD: 14.59%; and PD: 27.82%), and Bacteroidota (overall: 4.93%; HC: 8.44%; AD: 3.75%; and PD: 3.74%) (Fig. 3A). Firmicutes dominated across all groups but showed a decreasing trend from the HC group to the PD group. In contrast, Proteobacteria increased markedly in the PD group, whereas Bacteroidota showed a decreasing pattern in both AD and PD groups (Fig. 3A). Forest plot analysis showed that the relative abundance of Actinobacteriota was significantly higher in AD than in HC (*q* = 0.014), whereas Bacteroidota was significantly lower in AD than in HC (*q* = 0.014) (Fig. 3B). In contrast, no significant differences were observed between PD and HC after multiple testing correction for the predominant phyla (all *q* > 0.05). Although Proteobacteria appeared to be more abundant in PD than in HC, this difference did not reach statistical significance (*q* = 0.170).

To evaluate the potential influence of Abx exposure, the PD group was further stratified into children who had received Abx before stool collection (PD + Abx) and those who had not (PD − Abx) (Fig. 3C). Compared with HC, the PD + Abx subgroup showed a significantly higher relative abundance of Proteobacteria (*q* = 0.040), while Firmicutes, Actinobacteriota, and Bacteroidota did not differ significantly after multiple testing correction. A significant difference was also detected for the low-abundance phylum Campylobacterota (*q* < 0.001). In the PD − Abx subgroup, Bacteroidota was significantly lower than in HC (*q* = 0.006), whereas no significant differences were detected for Firmicutes, Actinobacteriota, or Proteobacteria. Significant differences were additionally observed for the low-abundance phyla Fusobacteriota, Desulfobacterota, and Verrucomicrobiota (all *q* < 0.001) (Fig. 3D).

**Figure 4.**
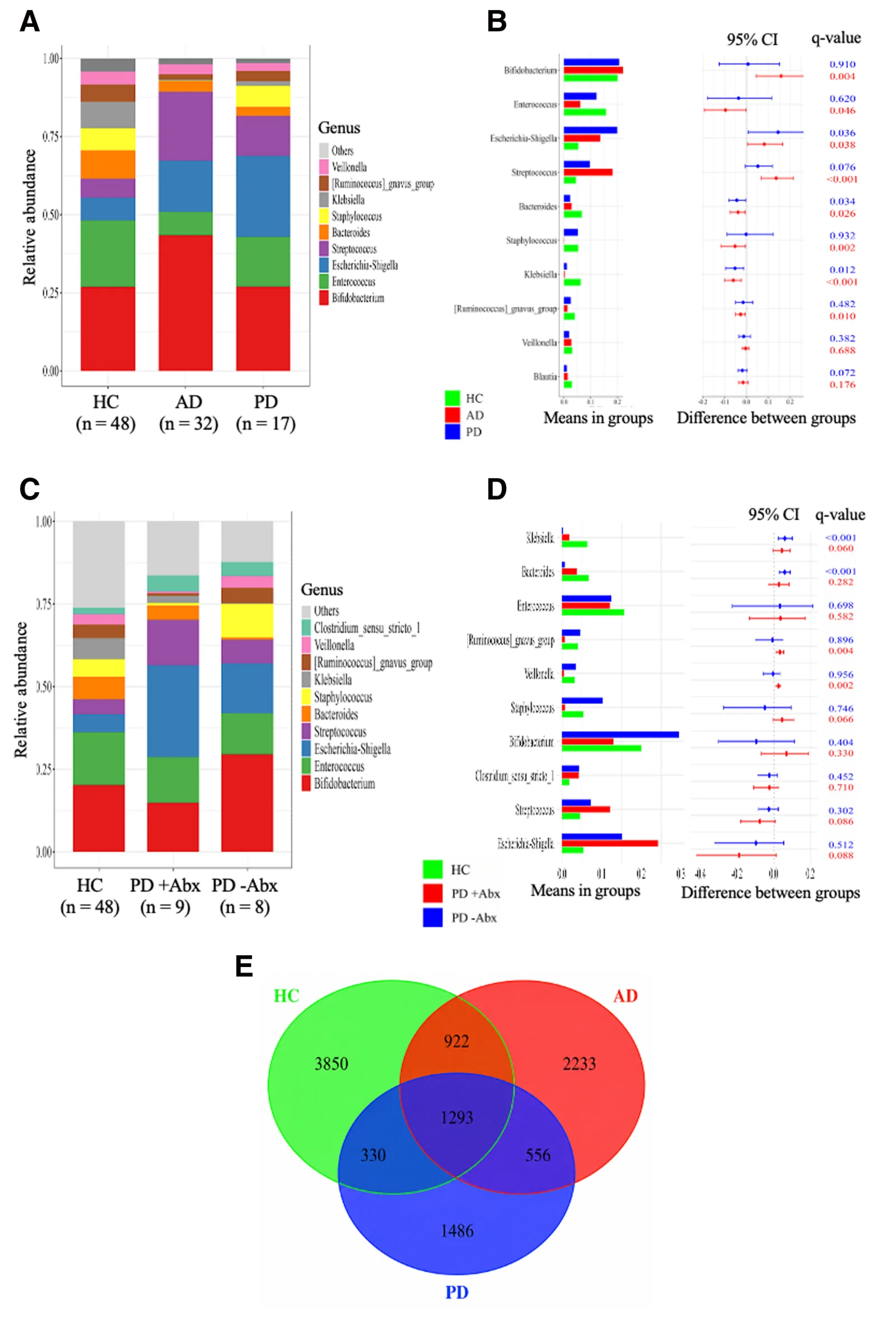
Genus-level composition of the gut microbiota and subgroup analysis according to antibiotic exposure. (A) Relative abundance of the predominant bacterial genera in healthy children (HC), children with acute diarrhea (AD), and children with persistent diarrhea (PD). (B) Mean relative abundance of the predominant bacterial genera in each group and forest plots showing the differences in relative abundance between AD and HC (red) and between PD and HC (blue), with 95% confidence intervals (95% CI) and Benjamini–Hochberg-adjusted *P* values (*q* values). (C) Relative abundance of the predominant bacterial genera in HC, children with persistent diarrhea who received antibiotics before stool collection (PD + Abx), and those who had not received antibiotics before stool collection (PD − Abx). (D) Mean relative abundance of the predominant bacterial genera and forest plots comparing PD + Abx and PD − Abx with HC, showing the differences in relative abundance with 95% confidence intervals (95% CI) and *q* values. (E) Venn diagram showing the numbers of shared and unique amplicon sequence variants (ASVs) among HC, AD, and PD. Abx = antibiotics.

At the genus level, the predominant bacterial taxa included *Bifidobacterium* (overall: 25.50%; HC: 19.96%; AD: 35.83%; and PD: 20.70%), *Escherichia–Shigella* (overall: 12.94%; HC: 5.37%; AD: 5.54%; and PD: 19.89%), *Enterococcus* (overall: 11.35%; HC: 15.75%; AD: 6.14%; and PD: 12.16%), *Streptococcus* (overall: 10.81%; HC: 4.53%; AD: 18.16%; and PD: 9.75%), and *Bacteroides* (overall: 3.94%; HC: 6.70%; AD: 2.84%; and PD: 2.28%) (Fig. 4A). Compared with HC, AD showed significantly higher relative abundances of *Bifidobacterium* (q = 0.004), *Escherichia–Shigella (q*** = **0.038), and *Streptococcus (q* < 0.001), but significantly lower relative abundances of *Enterococcus (q* = 0.046), *Bacteroides (q* = 0.026), *Klebsiella (q* < 0.001), and *Staphylococcus* (q** **= 0.046). In the PD group, *Escherichia–Shigella* was the only genus with a significantly higher relative abundance than in HC (*q* = 0.036) and was also the most abundant genus, whereas *Bacteroides (q* = 0.034), *Klebsiella (q* = 0.012), and the *Ruminococcus gnavus* group (*q* = 0.021) showed significantly lower relative abundances (Fig. 4B).

To evaluate the potential influence of Abx exposure, the PD group was stratified into children who had received Abx before stool collection (PD + Abx) and those who had not (PD − Abx) (Fig. 4C and D). Compared with HC, the PD − Abx subgroup showed significantly lower relative abundance of *Klebsiella (q* < 0.001) and *Bacteroides (q* < 0.001). By contrast, in the PD + Abx subgroup, significant differences from HC were observed for the *Ruminococcus gnavus* group (*q* = 0.004) and *Veillonella (q* = 0.002). No significant differences were observed in the relative abundance of other genera in the subgroup analysis. These findings show that the genus-level microbial profiles differed only partially between the PD + Abx and PD − Abx subgroups. Additionally, the Venn diagram demonstrated that the 3 groups shared 1293 amplicon sequence variants (ASVs), while 3850, 2233, and 1486 ASVs were unique to HC, AD, and PD, respectively (Fig. 4E), indicating shared and group-specific ASVs among the 3 groups.

**Figure 5.**
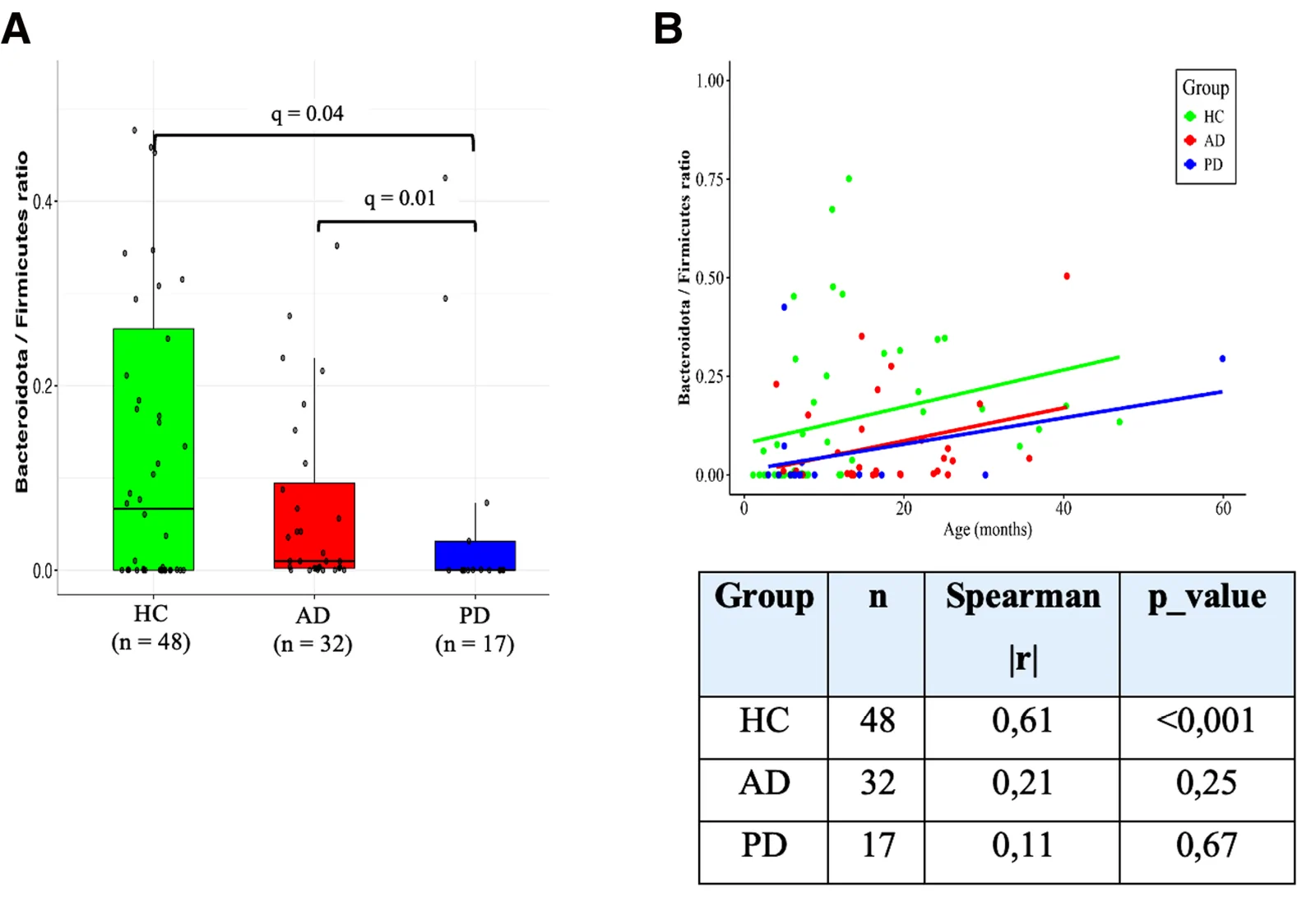
Bacteroidota/Firmicutes ratio and its association with age among healthy children (HC), children with acute diarrhea (AD), and children with persistent diarrhea (PD). (A) Comparison of the Bacteroidota/Firmicutes ratio among the 3 groups. Pairwise comparisons were performed using the Kruskal–Wallis test with Benjamini–Hochberg correction, and *q* values are shown above the box plots. (B) Association between age and the Bacteroidota/Firmicutes ratio in HC, AD, and PD. Scatter plots illustrate individual samples, and the accompanying table summarizes Spearman correlation coefficients (ρ) and corresponding *P* values for each group.

The Bacteroidota/Firmicutes ratio differed significantly among the study groups (Fig. 5A). Compared with HC, the ratio was significantly lower in both AD (*q* = 0.040) and PD (*q* = 0.010), whereas no significant difference was observed between AD and PD after multiple testing correction. Correlation analysis showed a significant positive association between age and the Bacteroidota/Firmicutes ratio in HC (Spearman ρ = 0.61, *P* < .001) (Fig. 5B). In contrast, no significant correlation was observed in either the AD group (ρ = 0.21, *P* = .25) or the PD group (ρ = 0.11, *P* = .67). These findings suggest that the positive association between age and the Bacteroidota/Firmicutes ratio observed in HC was not observed in children with diarrhea.

### 3.4. Differential taxonomic abundance among the 3 groups

**Figure 6.**
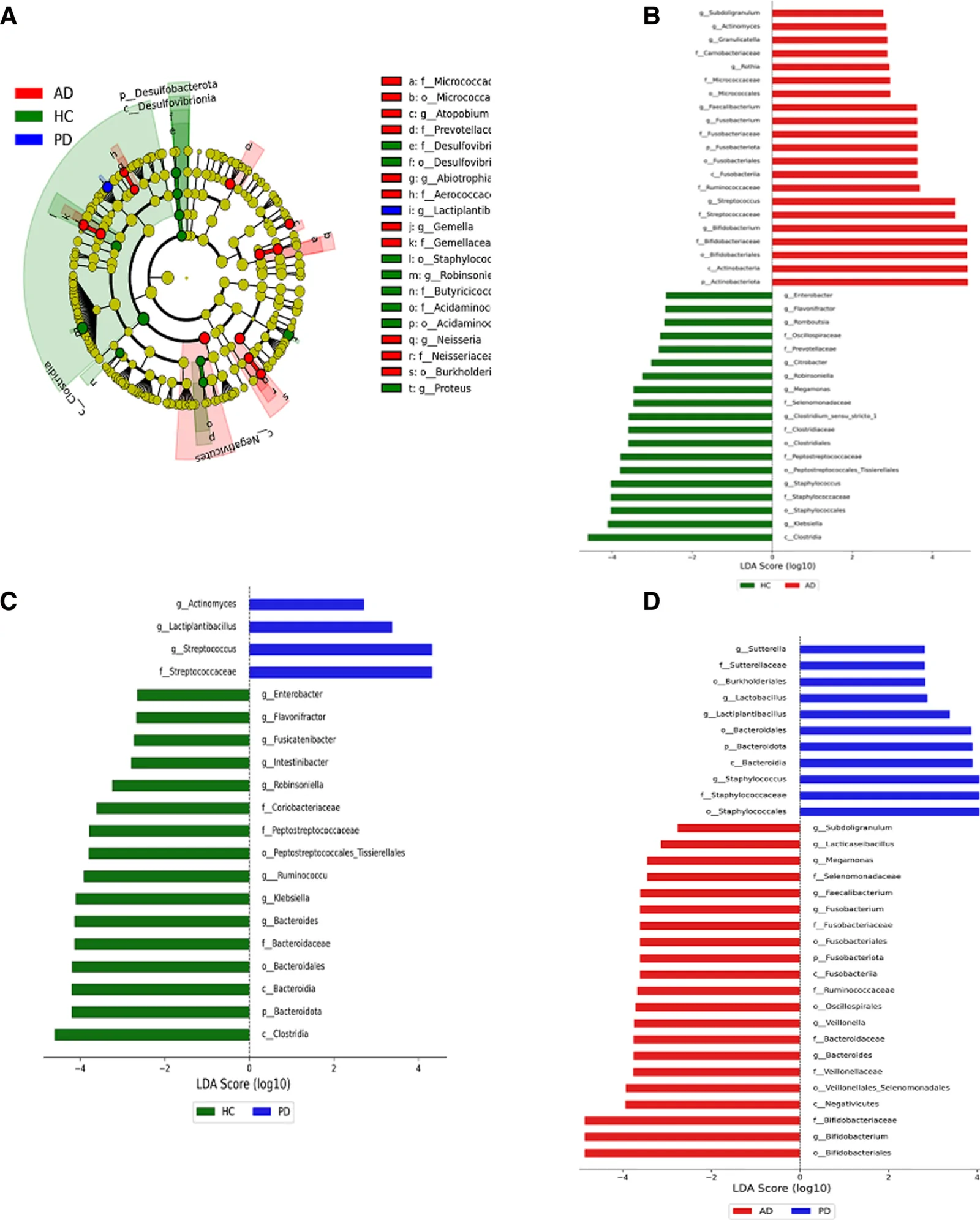
Linear discriminant analysis effect size (LEfSe) identifying differentially abundant bacterial taxa among healthy children (HC), children with acute diarrhea (AD), and children with persistent diarrhea (PD). (A) Cladogram showing the phylogenetic distribution of differentially abundant taxa. (B) LDA score plot comparing HC and AD. (C) LDA score plot comparing HC and PD. (D) LDA score plot comparing AD and PD. Only taxa with an LDA score greater than or equal to 2.0 are shown. LDA = linear discriminant analysis.

LEfSe analysis was performed to identify bacterial taxa that differed among HC, children with AD, and children with PD. A total of 304 discriminative taxa were identified, and taxa with an LDA score (log10) ≥ 2.0 were considered differentially abundant (Fig. 6). The cladogram illustrates the phylogenetic relationships of the discriminative taxa identified by LEfSe across the 3 study groups (Fig. 6A). Comparison between HC and AD revealed that taxa including Actinobacteriota, Fusobacteriota, *Bifidobacterium*, *Streptococcus*, and *Rothia* were enriched in the AD group, whereas Clostridia, *Klebsiella*, Staphylococcales, and Peptostreptococcaceae were enriched in HC (Fig. 6B). Comparison between HC and PD showed that Streptococcaceae, *Streptococcus*, *Lactiplantibacillus*, and *Actinomyces* were enriched in PD, whereas Bacteroidota, *Bacteroides*, *Klebsiella*, *Ruminococcus*, and Clostridia were enriched in HC (Fig. 6C). Direct comparison between AD and PD demonstrated distinct microbial signatures (Fig. 6D). The PD group was enriched in representative taxa such as Bacteroidota, Staphylococcaceae, *Staphylococcus*, *Lactobacillus*, *Lactiplantibacillus*, and *Sutterella*, whereas the AD group was enriched in *Bifidobacterium*, *Bacteroides*, *Veillonella*, *Faecalibacterium*, *Megamonas*, and *Subdoligranulum.* These findings indicate that acute and PD were associated with distinct gut microbial signatures.

## 4. Discussion

Understanding how gut microbiota changes during acute and persistent diarrhea is essential for developing effective strategies for the prevention and treatment of diarrheal diseases. By comparing the diversity and composition of the gut microbiota in children with AD, PD, and HCs, this study provides further insights into these microbial alterations. Overall, children with diarrhea exhibited reduced microbial diversity, distinct microbial community structures, and marked alterations in bacterial composition compared with HC. These alterations were already evident in AD, characterized by the enrichment of potentially pathogenic taxa together with the depletion of several commensal bacteria, and were more pronounced in PD, which showed the lowest microbial diversity and a more severe dysbiotic profile. These findings suggest that microbial dysbiosis observed during AD may persist in some children and could be associated with the dysbiotic profile observed in PD.

Our study demonstrated reduced gut microbial diversity in children with diarrhea, particularly among those with PD. Persistent intestinal mucosal injury, low-grade inflammation, and alterations in the intestinal environment, including changes in pH, nitric oxide, and redox status, may delay the reestablishment of gut microbial homeostasis.^[29]^ Supportively, a previous study showed that there might be a new, stable but less beneficial microbial ecosystem in PD.^[16,17]^ In the present study, subgroup analyses stratified by Abx exposure showed that significant reductions in Shannon diversity and Pielou evenness remained detectable only in the PD subgroup without prior Abx exposure. The findings suggest that several diversity alterations remained evident after stratification according to Abx exposure. These observations are consistent with previous studies suggesting that Abx exposure can alter gut microbiota composition and may delay microbiota recovery after AD.^[30,31]^ Reduced microbial diversity has been associated with disruption of the intestinal epithelial barrier and enhanced inflammatory responses.^[32]^

We found that gut microbiota composition in AD and PD was abnormal compared to controls. HC exhibited gut microbiota dominated by Firmicutes and Bacteroidota, whereas children with PD tended to exhibit a higher relative abundance of Proteobacteria together with a lower Bacteroidota/Firmicutes ratio. A previous study showed that an increase in the pro-inflammatory phylum Proteobacteria and a decrease in the Bacteroides/Firmicutes ratio were associated with bacteria-induced diarrhea.^[33]^ Furthermore, another study reported that a higher Firmicutes/Bacteroides ratio was associated with PD.^[17]^ This microbial signature is commonly associated with inflammatory dysbiosis and aligns with findings from previous studies.^[17,32]^ One possible explanation is that diarrhea may disrupt the intestinal mucus layer and epithelial barrier, creating a more oxygen-rich environment that favors the expansion of facultative anaerobic and pro-inflammatory bacteria.^[32]^ After stratification by prior Abx exposure, in addition to the overall trends, the PD + Abx subgroup showed an increased relative abundance of Proteobacteria, whereas the PD − Abx subgroup was characterized primarily by a reduced relative abundance of Bacteroidota. These findings suggest that prior Abx exposure may contribute to some of the alterations in gut microbiota composition observed in children with PD, although it is unlikely to fully explain the dysbiotic profile. Notably, an increased abundance of Proteobacteria has been associated with disruption of the intestinal ecosystem and is considered a hallmark of more severe gut microbial dysbiosis.^[34]^

Interestingly, we found that children with AD exhibited microbial alterations that were intermediate between the HC and PD groups. The gut microbiota in the AD group was characterized by increased relative abundances of *Escherichia–Shigella*, *Streptococcus*, and *Bifidobacterium*, along with decreased abundances of *Enterococcus, Bacteroides,* and *the Ruminococcus gnavus* group. A direct comparison between AD and PD further showed that AD was enriched in *Bifidobacterium*, *Bacteroides*, *Veillonella*, and *Faecalibacterium*. These findings suggest that although the gut microbiota in children with AD had already shifted toward a dysbiotic state, beneficial genera remained more abundant than in PD, indicating a less severe degree of microbial disruption. Consistently, a previous study showed that even short-term diarrhea can reduce microbial diversity and allow opportunistic bacteria to proliferate.^[35,36]^ Similarly, children with PD in our study exhibited enrichment of bacterial taxa previously associated with intestinal dysbiosis or inflammation, accompanied by depletion of taxa generally regarded as beneficial, in agreement with observations by Tesfaw^[16]^ and Pham.^[17]^ Likewise, Pop^[37]^ demonstrated that childhood diarrhea is associated with large-scale alterations in gut microbiota composition, whereas Subramanian^[10]^ showed that in undernourished children, recovery of the gut microbiota remains incomplete, with persistent microbiota immaturity despite nutritional rehabilitation. Notably, previous studies showed that *Streptococcus* is enriched during early stages of AD.^[12,13,36]^ The recovery process from diarrhea is characterized by declining abundances of *Streptococcus* and *Rothia*, accompanied by the restoration of *Bacteroides*, Lachnospiraceae, and Ruminococcaceae.^[30,38]^ In our study, *Bacteroides* remained depleted in PD, whereas LEfSe analysis identified *Streptococcus* as a discriminative taxon despite the absence of a significant difference in its relative abundance compared with HC in the genus-level abundance analysis. This pattern may suggest that gut microbial dysbiosis persists from AD into PD in some children, although this hypothesis cannot be confirmed by our study. Our subgroup analyses further showed that some genus-level alterations remained evident after stratification by Abx exposure, whereas others were no longer statistically significant. These findings suggest that prior Abx exposure may account for some of the observed alterations in gut microbiota composition but is unlikely to fully explain the dysbiotic profile associated with PD. Overall, the observed differences in bacterial composition are consistent with evidence that persistent enrichment of potentially harmful bacteria is associated with PD.^[16,17]^ However, because of the cross-sectional design, the direction of this relationship and any causal role of gut microbiota alterations cannot be determined from this study.

## 5. Limitations

This study has several limitations. First, the sample size is small. Additional samples are needed to validate the findings. Second, some children in the PD group had received Abx or probiotics prior to hospital admission, which may have influenced gut microbiota composition and acted as a potential source of residual confounding. Although an additional subgroup analysis was performed to evaluate the potential impact of prior Abx exposure, residual confounding cannot be completely excluded. Additionally, the cross-sectional study design prevents the establishment of causal relationships between variables. Another limitation is the lack of conventional microbiological and virological testing, which precluded the identification of specific etiologies of diarrhea and limited our ability to determine whether the observed microbiota alterations were attributable to particular infectious pathogens. Therefore, some of the observed microbiota differences between AD and PD may have been influenced by differences in the etiologies of diarrhea rather than diarrhea duration alone.

## 6. Conclusion

This study showed that gut microbiota composition and diversity differed among HC and those with AD or PD. Both acute and PD were associated with distinct microbial alterations, with more pronounced changes observed in PD. These findings provide insights into gut microbiota characteristics associated with pediatric diarrhea and support the need for future longitudinal studies incorporating comprehensive pathogen identification to clarify temporal relationships, evaluate the potential of microbiome-based biomarkers, and explore the role of microbiota-targeted interventions in PD. Our findings should therefore be interpreted with caution and require validation in future studies incorporating comprehensive pathogen identification.

## Acknowledgments

The authors would like to express their sincere gratitude to the University of Medicine and Pharmacy at Ho Chi Minh City; Can Tho University of Medicine and Pharmacy; Children’s Hospital 1, Ho Chi Minh City; and Gene Solutions. During the preparation of this work, the authors used ChatGPT to improve the language and readability of the manuscript. After using this tool, the authors carefully reviewed and revised the content as needed and take full responsibility for the final version of the manuscript.

## Author contributions

**Conceptualization:** Nghi Huu Chung, Tuan Anh Nguyen.

**Data curation:** Nghi Huu Chung.

**Formal analysis:** Nghi Huu Chung.

**Investigation:** Nghi Huu Chung.

**Methodology:** Nghi Huu Chung, Tuan Anh Nguyen, Van Hung Pham, Luan Thanh Nguyen, Nhan Trong Phan, Chuyen Quoc Vo, Nguyen Thanh Nhu.

**Software:** Nghi Huu Chung.

**Validation:** Tuan Anh Nguyen, Nguyen Thanh Nhu.

**Visualization:** Nghi Huu Chung, Van Hung Pham, Luan Thanh Nguyen, Nhan Trong Phan, Chuyen Quoc Vo.

**Writing – original draft:** Nghi Huu Chung.

**Writing – review & editing:** Tuan Anh Nguyen, Nguyen Thanh Nhu.
